# Chlamylipo, a *Chlamydomonas*-in-liposome microswimmer: Self-propelled swimming and associated lipid membrane flow

**DOI:** 10.2142/biophysico.bppb-v23.0019

**Published:** 2026-05-26

**Authors:** Shunsuke Shiomi, Koichiro Akiyama, Hiromasa Shiraiwa, Sota Hamaguchi, Daiki Matsunaga, Tomoyuki Kaneko, Masahito Hayashi

**Affiliations:** 1 Department of Frontier Bioscience, Graduate School of Science & Engineering, Hosei University, Koganei, Tokyo 184-8584, Japan; 2 Graduate School of Engineering Science, The University of Osaka, Toyonaka, Osaka 560-8531, Japan; 3 Department of Biotechnology and Life Science, Tokyo University of Agriculture and Technology, Koganei, Tokyo 184-8588, Japan

**Keywords:** *Chlamydomonas reinhardtii*, liposome, membrane flow, microswimmer, biohybrid robots

## Abstract

Developing active transport systems for micro cargo delivery is challenging because it requires overcoming the constraints imposed by low Reynolds numbers. We developed a bio-hybrid micro-swimmer, “Chlamylipo” consisting of the green alga *Chlamydomonas reinhardtii*, encapsulated within a giant liposome. Although internal encapsulation offers cargo protection, it necessitates a mechanism to transmit the propulsion force across a closed membrane. We demonstrated that Chlamylipo exhibited forward swimming and phototactic directional control. High-speed imaging of the membrane shape and fluid flow revealed that the driving force originated from periodic membrane deformations and was accompanied by characteristic fluid dynamics. Flow analysis showed rapid oscillations at tens of hertz corresponding to flagellar beating, superimposed on slower axial migration at approximately 4 Hz, associated with cell rotation. Corresponding flow signatures were also detected in the external fluid, indicating mechanical coupling of the lipid bilayer. Membrane domain tracking further revealed that the fluid motions inside and outside the membrane were coupled through viscous friction and membrane deformation, generating a characteristic four-vortex flow field consistent with a two-point force model. Collectively, these results suggest that membrane flow primarily reflects force transmission across the bilayer, whereas forward propulsion is primarily driven by periodic membrane deformation. This study elucidates the physical mechanism of force transmission in encapsulated swimmers, demonstrating that internal hydrodynamic power can effectively drive the motion of microscopic containers.

## Significance

The development of autonomous micro-swimmers for targeted drug delivery is a major challenge in the field of biophysics. We present “Chlamylipo,” a hybrid system in which a swimming alga is encapsulated within a lipid vesicle. This study is significant because it demonstrates that an enclosed swimmer can propel a microscopic container solely via hydrodynamic coupling across a closed membrane, without direct external mechanical links. Furthermore, we achieved external directional control using phototaxis. This study provides physical insights into fluid-membrane interactions and proposes a novel strategy for designing light-guided active transport carriers.

## Introduction

Liposomes are lipid bilayer vesicles capable of encapsulating molecules, particles, and other materials of varying sizes and properties. Due to this versatility, they have been extensively utilized as model cell-sized compartments and carriers for delivery applications [1,2]. Numerous previous studies have suggested passive strategies for liposome transport based on external flow, chemical gradients, and interactions with the surrounding environment. However, in recent years, active transport systems that integrate cargo carriers with motile microorganisms or other active agents have garnered increasing attention as a strategy for transporting microscopic objects [3–5]. For instance, soft erythrocyte-based bacterial microswimmers have been developed by coupling *Escherichia coli* to red blood cells through biotin–avidin binding, thereby leveraging bacterial motility for cargo transport [6]. In another example, bacteria encapsulated within lipid vesicles were shown to deform the vesicle membrane and drive the active transport of the entire vesicle [7]. These studies highlight the potential of microorganisms as active propulsive agents for soft, microscopic carriers.

In this study, we developed a microorganism-driven liposome system, termed Chlamylipo, by encapsulating *Chlamydomonas reinhardtii* inside lipid vesicles and analyzed its swimming behavior. *Chlamydomonas* is a unicellular green alga that swims using two flagella. The flagellar beat consists of a power stroke, moving the flagella from front to back, and a recovery stroke, moving them back to front. Its eyespot senses light in all directions during self-rotation. By adjusting flagellar beating in response to light changes, *Chlamydomonas* achieves phototaxis, altering its swimming direction toward or away from light [8–12].

In microorganisms, swimming dynamics differ from those of macroscale organisms. The Reynolds number (Re) indicates the types of swimming dynamics, representing the ratio of inertial to viscous forces. For *Chlamydomonas*, Re is typically on the order of 10^–3^ to 10^–2^, indicating that viscous forces dominate [13]. In low-Re environments, organisms cannot achieve net movement through reciprocal motion, known as the scallop theorem [14]. However, the time-reversal asymmetric motion of the flagella enables *Chlamydomonas* to swim forward. These features make *Chlamydomonas* a propulsive engine for transporting artificial micro-objects and synthetic carriers [15,16].

Building on these prior applications, we demonstrate that Chlamylipo exhibits forward swimming and phototaxis. By analyzing membrane deformation during swimming, we confirmed that the liposome membrane also undergoes time-reversal asymmetric deformation, providing a basis for the propulsion of the entire liposome. We also identified characteristic membrane flow patterns and showed that their main features can be reproduced using a two-point force model representing flagellar action. Our results provide fundamental insights into how asymmetric beating inside a membrane compartment generates propulsion through fluid coupling. The potential use of such systems for active transport or drug delivery remains an intriguing subject for future studies.

## Materials and methods

### Chemicals

The phospholipids 1,2-dioleoyl-*sn*-glycero-3-phosphocholine (DOPC), 1,2-dipalmitoyl-*sn*-glycero-3-phosphocholine (DPPC), 1,2-diphytanoyl-*sn*-glycero-3-phosphocholine (DPhPC), and 1,2-distearoyl-*sn*-glycero-3-phosphoethanolamine-N- (lissamine rhodamine B sulfonyl) (ammonium salt) (RhPE) were purchased from Avanti Polar Lipids (USA). DOPE and ATTO 488-DOPE (488 PE) were purchased from ATTO-TEC. DOPC was used because of widespread use in liposome preparation. A mixture of DPhPC, DPPC, and cholesterol was used to generate phase-separated membrane domains for flow visualization. MCT oil (Nisshin OilliO) was purchased from supermarkets. Phospholipids dissolved in chloroform (10 mM or 10 μM) were stored at –20°C. MCT oil was degassed at 1.2 kPa for 30 min, flushed with nitrogen, and stored in the dark at room temperature. All other chemicals were purchased from Wako, TCI, or Merck.

### Cultivation of *Chlamydomonas*

Wild-type (CC124 and CC125), paralyzed flagella mutant (*pf18*), and impaired flagellar autotomy mutant (*fa1*) of *Chlamydomonas reinhardtii* were gifted by Prof. Masafumi Hirono (Hosei University). *Chlamydomonas* was cultured in Tris-acetate phosphate (TAP) medium [17] at 26°C with aeration and 12 h/12 h light/dark cycles.

### Preparation of lipid solutions

Phospholipids dissolved in chloroform were mixed in a PCR tube, and the total lipid content in the tube was set to 100 nmol. The lipid composition was set to DOPC:488 PE=1:0.01 (molar ratio). Chloroform was completely evaporated in a desiccator at 1.2 kPa for 30 min. After adding 100 μL of MCT oil to the dried lipids, the mixture was heated at 70°C whilst inverting and vigorously tapping the tube every 5 min to obtain a clear 1 mM solution. PCR tubes containing lipid solutions were stored in a dry atmosphere with silica gel in the dark at room temperature and used within six months. Before use, lipid precipitation was checked using phase-contrast and fluorescence microscopy. To visualize membrane flow using membrane phase separation, the lipid composition was set to DPhPC:DPPC:488 PE=0.5:0.5:0.01 (mol), and 100 μM water-soluble cholesterol was added to the external solution before observation, inducing the formation of phase separation domains in the membrane.

### Encapsulation of *Chlamydomonas* into giant unilamellar vesicles

*Chlamydomonas* cells were encapsulated into giant unilamellar vesicles using the water-in-oil emulsion-transfer method with centrifugation [18,19]. Briefly, cultured cells in the logarithmic growth phase were harvested and resuspended in an inner solution (100 mM sucrose, 30% Percoll, and 1 mg/ml BSA) at 1.5×10^7^ cell/ml. Next, 2 μl of the inner solution was added to 20 μl of the lipid solution in a PCR tube. The tube was agitated vigorously by sliding it on a tube stand several times to obtain a whitish homogeneous emulsion. The entire emulsion was placed on 30 μl of outer solution (100 mM glucose, 1 mg/ml BSA) in another PCR tube. The tube was centrifuged at 500×G and 20°C for 5 min to generate Chlamylipos and precipitate them at the bottom of the tube. The oil layer was removed, and the precipitate was collected with the outer solution and resuspended in a new tube by tapping.

### Microscope imaging

The motion of Chlamylipos was observed using phase contrast, dark field, and epifluorescent microscopes (BX53 and IX71, Olympus, Japan) equipped with a 40x objective lens. Simultaneous observation of the autofluorescence of the cell body (red) and fluorescence of the liposome membrane (green) was achieved using a wide-pass dichroic mirror unit (U-FBW, Olympus, Japan). The images were captured using a high-speed CMOS camera (DFK33UX287 for color and DMK33UX287 for monochrome imaging; The Imaging Source, Germany) at 30, 300, or 600 fps and recorded directly on the HDD as uncompressed video files.

### Phototaxis experiment

An inverted microscope (IX71) was used to control the movement direction of the cells via phototaxis. A green LED with a wavelength of 525 nm (L3-G2530-12500, FULL SUN OPTOTECH, Taiwan) served as the induction light, positioned on the microscope stage with the samples sandwiched and facing each other. To prevent *Chlamydomonas* from being attracted to the observation light owing to phototaxis, a long-pass filter (RG630, SCHOTT, Germany) with a transmission cutoff wavelength of 630 nm was placed above the condenser, and only light with wavelengths that did not induce phototaxis was used for the observation.

### Visualization of the flow field of the liposome membrane

Color videos of Chlamylipo with phase-separated membrane domains at 300 fps were split into RGB channels; the R-channel images were analyzed to track the motion of the cell body, and the G-channel images were analyzed to obtain the flow field of the liposome membrane. The centroid coordinates of the cell body were automatically measured using the ImageJ software, whereas those of the phase-separated domains were determined manually. The north-south axis was defined as the swimming axis, which was calculated as the first principal component of the entire trajectory of the centroid of the cell body, and the latitude and longitude were calculated based on this axis.

### Simulation model of the membrane flow field of Chlamylipos

We utilized a theory [20] to understand the relationship between the velocity field on the liposome membrane and flagella beating force. Here, we briefly explain the equations used in the manuscript and, the assumptions underlying their derivation. Consider a spherical liposome with a membrane viscosity $\eta_{m}$ and radius *R*. The fluids inside and outside the membrane are incompressible Newtonian fluids with viscosities $\eta^{+}$ and $\eta^{-}$, respectively. The Stokes equations that describe the velocity fields of the membrane $\nu^{m}$ and the interior (–)/exterior (+) fluids v are given as

(1) $$\eta_{m}\Delta v^{m}=\nabla p_{m}$$

(2) $$\eta^{\pm }\Delta v^{\pm }=\nabla p^{\pm }$$

where $p_{m}$ and $p^{\pm }$ are the pressures of the membrane and interior/exterior fluids, respectively. By assuming velocity continuity and stress balance conditions at the membrane, the velocity fields can be solved analytically when there is no deformation of the membrane. When a point force $F_{y}$=(0, *F*, 0) is applied to the north pole (0, 0, *R*) of the spherical membrane, the velocity field at position (*R* sin *θ* cos *ϕ*, *R* sin *θ* sin *ϕ*, *R* cos *θ*) is given [20,21] as:

(3) $$v_{np}^{m}\left(R,\theta ,\phi \right)=F\left[\sin\;\phi \;cosec\theta S_{1}\left(R,\cos\;\theta \right)\hat{\theta }+\cos\;\phi \;\left\{cot\;\theta \;S_{1}\left(R,\cos\;\theta \right)+S_{2}\left(R,\cos\;\theta \right)\right\}\hat{\phi }\right]$$

(4) $$S_{n}\left(R,\cos\;\theta \right)=\frac{1}{4\pi \eta_{m}}\sum_{l=1}^{\infty }\frac{2l+1}{l\left(l+1\right)}\frac{1}{s_{l}}P_{l}^{n}\left(\cos\;\theta \right)$$

(5) $$s_{l}=l\left(l+1\right)-2+\frac{R\eta^{-}}{\eta_{m}}\left(l-1\right)+\frac{R\eta^{+}}{\eta_{m}}\left(l+2\right)$$

where $\hat{\theta }$ and $\hat{\phi }$ are unit vectors in *θ* and ϕ directions, respectively, and $p_{l}^{n}$ is the associated Legendre polynomial.

### Simulation model of the membrane flow field of Chlamylipos

The membrane velocity resulting from the beating of the two flagella can be represented by two-point forces on a spherical liposome membrane. We evaluated the exerted forces *F* and the polar angle at which the force was exerted, $\theta_{f}$, via the fitting process.

The velocity field of the membrane was evaluated by tracking lipid domains. Because the liposome radius and the viscosities ($\eta^{+}$ and $\eta^{-}$) are known, we can estimate the force magnitude *F* and polar angle $\theta_{f}$, which is the representative position where the force is exerted, from the least-squares fitting process. Because Equations (3)–(5) are solutions under the linear Stokes equation, the velocity field owing to the two-point forces can be evaluated by simply superimposing two velocity fields. By setting the force magnitude *F* and the polar angle of the two forces $\theta_{f}$ as the fitting parameters, we obtained these parameters by minimizing the error between the estimated and experimentally obtained velocities. The parameters utilized in the fitting process are as follows: *R*=10 μm, $\eta^{m}$=6.5×10^–8^ Pa·s·m [21], $\eta^{-}$=1.63×10^–3^ Pa·s, and $\eta^{+}$=0.99×10^–3^ Pa·s. The obtained force magnitude is *F*=8.13×10^–11^ N and the polar angle $\theta_{f}$=1.41 rad from the North Pole.

## Results

### Encapsulation of *Chlamydomonas* in giant unilamellar vesicles

**Figure 1:**
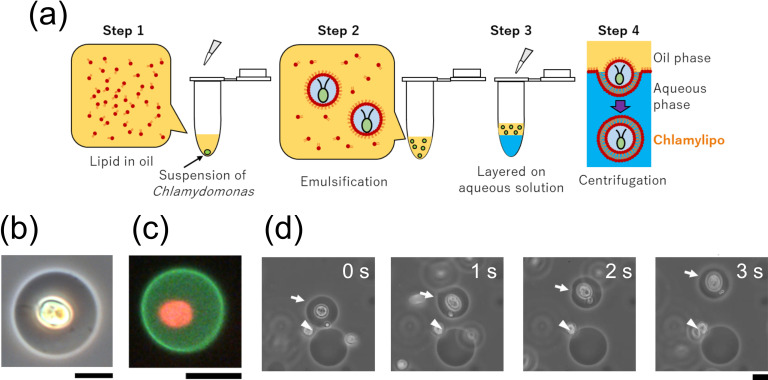
Preparation of Chlamylipos: giant liposomes containing a living *Chlamydomonas*. (a) Liposomes were obtained by creating a W/O emulsion with an aqueous phase containing a phospholipid solution and *Chlamydomonas*, layered in an outer solution, and centrifuged. (b) Phase-contrast images of the produced Chlamylipos (color photography). (c) Fluorescence image of a Chlamylipo. The liposome membrane appeared green, while the chloroplast’s autofluorescence was seen as red. (d) Time-lapse phase contrast images over three seconds of swimming Chlamylipo. Liposomes without encapsulated cells did not move (indicated by an arrowhead), whereas Chlamylipo moved approximately 20 μm at a constant speed in one direction over three seconds (indicated by an arrow). All scale bars represent 10 μm.

In the encapsulation process of *Chlamydomonas* using the liposome and water-in-oil (W/O) emulsion template method, *Chlamydomonas* was successfully encapsulated within liposomes (Fig. 1a). When observed under phase contrast, the green *Chlamydomonas* cell body was visible inside the gray liposomes (Fig. 1b). When the liposomes were observed using blue-excited green fluorescent phospholipids, both the green fluorescence from the liposome membrane and the red autofluorescence derived from chlorophyll in the cell body were observed simultaneously (Fig. 1c). In all cases, the encapsulated cells continued to move, even when surrounded by liposomes. By adjusting the internal fluid with the addition of 30% Percoll to match the density of *Chlamydomonas*, the encapsulation efficiency improved to 100.2±14.5 Chlamylipos/μL were achieved. In 94% of the Chlamylipos, a single cell was encapsulated, and 22% of Chlamylipos travelled more than twice the diameter of the liposome in 30 s while remaining enclosed in the liposome (Fig. 1d). The swimming speed of Chlamylipo varied greatly at 8.0±1.9 μm/s (Fig. S1). Furthermore, no clear correlation was observed between swimming speed and liposome diameter (Fig. S2). The swimming speed is likely to be influenced not only the liposome diameter but also the length of membrane protrusion and beat frequency of flagella. Improvement of high-speed recording of the fluorescence images of Chlamylipo’s membrane will reveal the relation between these parameters underlying its propulsion mechanism. Although the *Chlamydomonas* inside exhibited normal flagellar movement and self-rotation, 49% of the Chlamylipos did not move forward. The lack of motility was due to insufficient flexibility of the liposome membrane, as discussed in the following section “Periodic membrane deformation of Chlamylipo.” Chlamylipos prepared with the *Chlamydomonas pf18* mutant strain, which lacks flagellar motility, neither swimming nor rotation was observed (0.08±0.05 μm/s, Movie S1). A small number of liposomes encapsulating more than two cells were observed but coordinated movement of two cells was not observed, and stable swimming was not observed (Movie S2).

### Phototactic response of Chlamylipo

**Figure 2:**
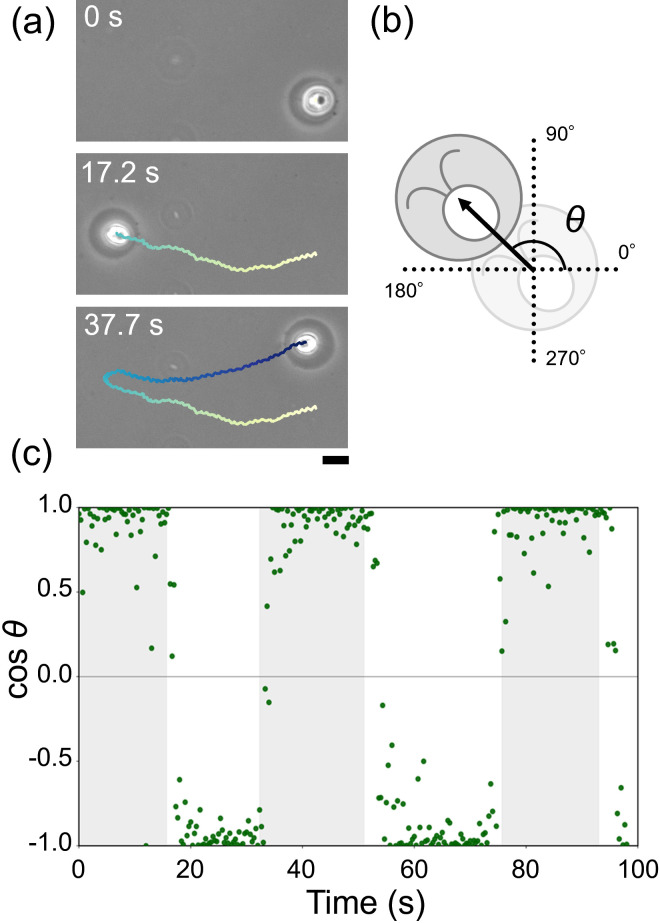
Swimming and Phototactic Direction Control of Chlamylipo. (a) Phase-contrast images tracing the movement of Chlamylipo. The green light was continuously illuminated from the left side of the image until 17.2 s and from the right side until 37.7 s, resulting in a change in the movement direction. Scalebar represents 10 μm. (b) Cosine evaluation image diagram of the Chlamylipo direction. (c) Change in the direction of Chlamylipo movement. In areas with a gray background, green light was shone from the 0-degree direction (cos *θ*=1.0), and in areas with a white background, light was shone from the 180-degree direction (cos *θ*=–1.0).

The photoreceptors involved in phototaxis in *Chlamydomonas* are known to be highly sensitive to blue to blue-green wavelengths [8,9]. In this study, green light, which is close to this wavelength range and commonly used in phototaxis studies [10], was used as the attractant light. When green light shone from the side during the observation of Chlamylipo, it swam in the direction closer to (or further from) the light source (Fig. 2a). Subsequently, when green light shone from the opposite direction, the *Chlamydomonas* inside changed direction and swam in the opposite direction within a second (Fig. 2, Movie S3). This directional change was sustained for over 16 cycles, covering a total distance of approximately 1.2 mm (Movie S4). Furthermore, even in Chlamylipos that did not swim, the encapsulated *Chlamydomonas* cells repeatedly changed direction in response to green light.

### Periodic membrane deformation of Chlamylipo

**Figure 3:**
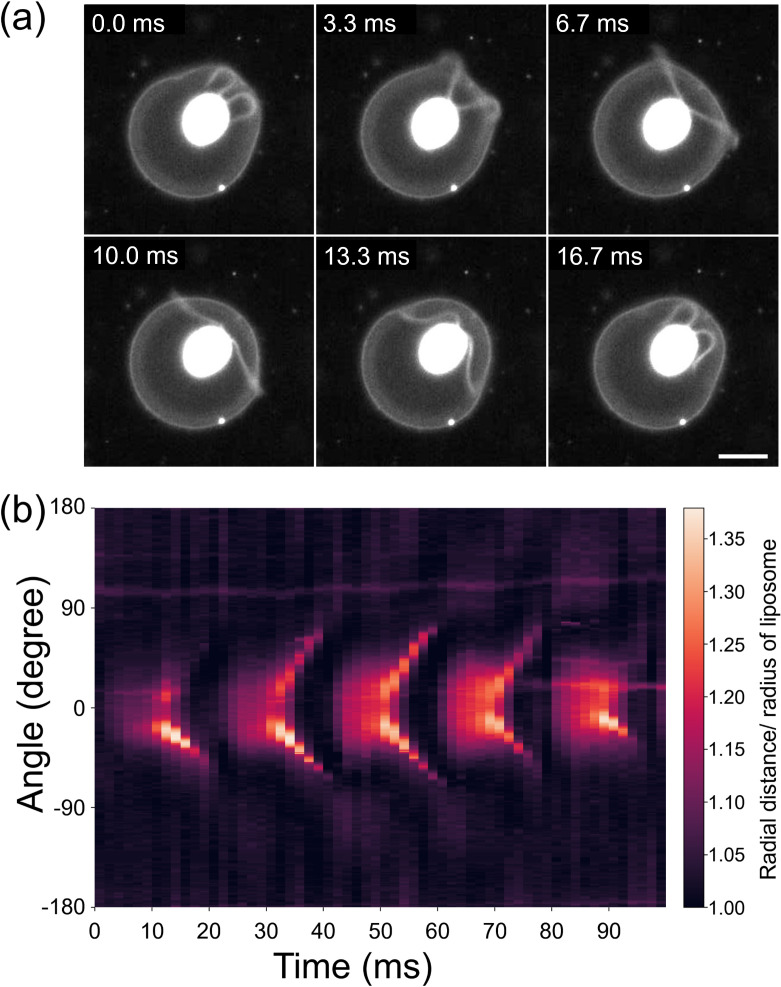
Periodic membrane deformation of a Chlamylipo. (a) Superimposed image sequence of a Chlamylipo recorded at 600 fps using dark-field (cell body and flagella) and fluorescence (membrane) microscopy. Scalebar represents 10 μm. (b) Kymograph of the periodic membrane deformation of a Chlamylipo. The radial distance of the membrane elements relative to the forward direction is color-coded.

When the liposome membrane was labeled with fluorescence and recorded at a high frame rate (600 fps) using dark-field microscopy while applying excitation light, periodic membrane deformations synchronized with flagellar beating were observed (Fig. 3, Movie S5). *Chlamydomonas* swims with its flagella leading; accordingly, we define the “front” as the flagella-leading side and the “rear” as the opposite side of the cell. At the beginning of the effective stroke, two membrane protrusions were formed at the front as the flagella pushed the liposome membrane outward. As the effective stroke progressed, the membrane protrusions moved from the front to the left and right sides. At the end of the effective stroke, as the flagellar tips were drawn close to the cell body, the membrane protrusions shortened and vanished. During the recovery stroke, no membrane protrusions were observed, and new membrane protrusions were formed at the beginning of the next effective stroke cycle. Because the plane containing the two flagella (flagellar plane) rotated around the anterior-posterior axis in conjunction with the internal rotation of *Chlamydomonas*, the length of the membrane protrusions periodically increased and decreased on the kymograph (Fig. 3b). Note that 0° in the kymograph corresponds to the direction of the *Chlamydomonas*. In Chlamylipos that did not form membrane protrusions associated with flagellar movement, forward swimming was not observed.

### Coupled flow inside and outside the liposome

**Figure 4:**
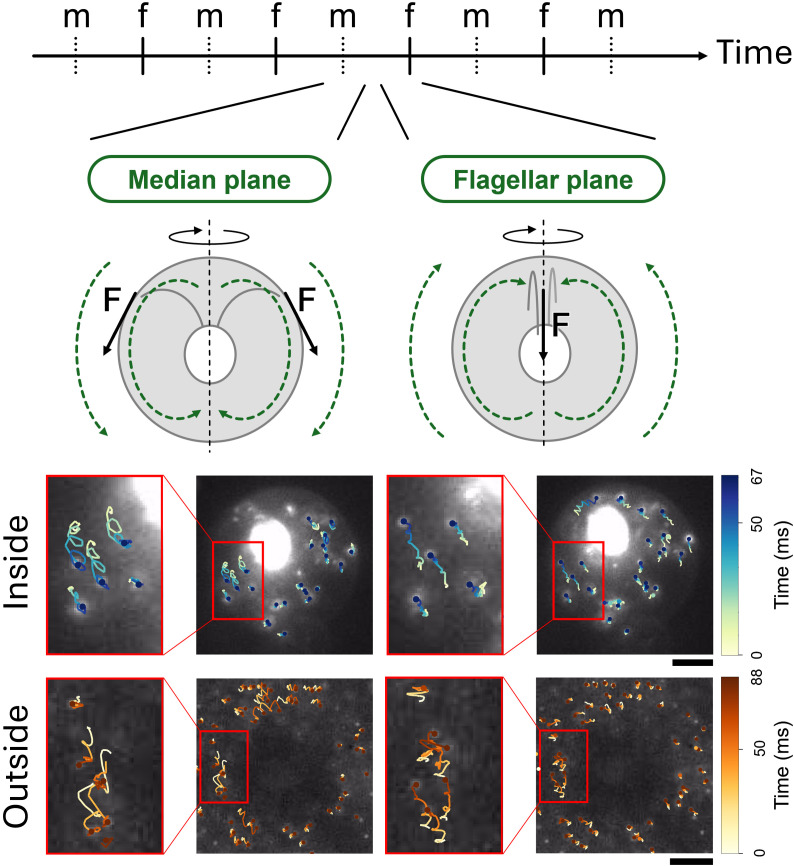
Fluid dynamics of internal and external fluids in Chlamylipo. As *Chlamydomonas* rotates while swimming, Chlamylipo appears to alternate between two orientations when viewed perpendicular to the swimming direction: the median plane (m-plane), where the cell body is viewed from the front, and the flagellar plane (f-plane), where the flagellar beat is viewed from the side (top panels). Middle panels: Observation of tracer beads inside the liposome. The beads exhibited small and rapid circular motions at several tens of hertz. In the m-plane view, beads flow from anterior to posterior, whereas in the f-plane view, they flow from posterior to anterior. Bottom panels: Similarly, the tracer beads outside the liposome exhibited small and rapid circular motions. Beads flow from anterior to posterior in the m-plane view and from posterior to anterior in the f-plane view. The brightness of the video was adjusted to visualize the particle trajectories. Trajectories were drawn from 0 to 66.7 ms (m-plane) and 66.7 to 133.3 ms (f-plane) for the internal fluid and from 0 to 83.3 ms (m-plane) and 83.3 to 166.7 ms (f-plane) for the external fluid. Scalebars represent 10 μm.

The flow of liquid inside and outside the Chlamylipo was visualized by adding tiny fluorescent beads to either the internal or external fluid (Fig. 4). The movement of fluorescent beads within a thickness of 1 μm, which is the focal depth of the objective lens, was observed. The tracer particles inside the Chlamylipo exhibited small, rapid circular motions at several tens of hertz, while also moving back and forth along the body axis with a period of approximately 4 Hz (Movie S6–8). Similarly, the tracer particles outside the Chlamylipo also showed small, rapid back-and-forth motions at several tens of hertz and moved back and forth along the body axis with a period of approximately 4 Hz (Movie S9–11).

### Visualization of membrane dynamics using phase-separated domains

**Figure 5:**
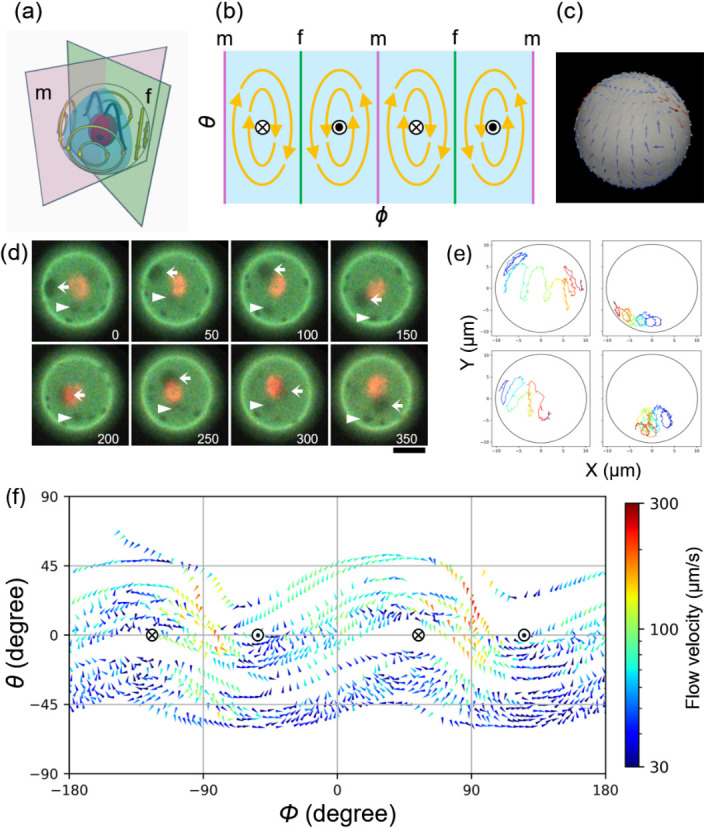
Membrane flow field on a Chlamylipo. (a) Schematic diagram of a Chlamylipo. The yellow arrows indicate the flow directions of the four predicted vortices. Plane “f” contains two flagella of a *Chlamydomonas*, and plane “m” indicates a median plane perpendicular to the “f” plane. (b) Flat map of the membrane flow field as shown in (a). Vertical lines indicate the cutting lines on the membrane by the “f” and “m” plane in (a). The centers of the four vortices are marked by ⊙ and ⊗. (c) Incompressible 2D spherical field with two-point forces. The position and orientation of the forces are consistent with *Chlamydomonas* flagella in (a). (d) Sequential images of a self-rotating Chlamylipo. The cell body (red) and liposome membrane (green) with phase-separated domains (black dots) are shown. Elapsed times are shown in ms. Scalebar represents 10 μm. (e) Typical trajectories of phase separated domains for 1 s. The time progressed from blue to red. (f) Membrane flow field in the coordinate system rotating with *Chlamydomonas* of a Chlamylipo. The centers of the four vortices are marked by ⊙ and ⊗ as shown in (b).

Let us consider the membrane flow generated in the liposome by the movement of *Chlamydomonas* inside a Chlamylipo (Fig. 5a). The solution flow produced by the repeated effective and recovery strokes of the flagella can be approximated as a long-term average by two steady backward flows near the flagella. The membrane flow induced by the flagella causes the fluid inside the liposome to flow, dragging the liposome membrane. Because the internal fluid is confined within the spherical liposome, the backward flagellar flow toward the rear of the cell body must always be accompanied by a return flow toward the front of the cell body. Furthermore, because the liposome membrane is a two-dimensional incompressible fluid on a closed surface, the backward membrane flow generated near the origin of the flagellar flow must also be accompanied by a return flow toward the front of the cell body. The two flagellar flows generated on the flagellar plane “f,” which contains the two flagella, are accompanied by two return flows toward the front on the median plane “m” perpendicular to it. Consequently, four vortices are expected to form on the liposome membrane (Fig. 5a, b). Even in the theoretical model in which point forces were applied at two points on the spherical membrane, the formation of four vortices was predicted (Fig. 5c). In reality, as *Chlamydomonas* rotates, it is thought that the four vortices also move in the equatorial direction. To visualize the membrane flow occurring on the Chlamylipo, it was fabricated with a lipid composition that resulted in two-dimensional phase separation on the membrane (Fig. 5d, Movie S12). Multiple black spots representing phase-separated domains were observed on the green fluorescent liposome membrane, while the *Chlamydomonas* cell body rotated at a frequency of 2.2 Hz. Each domain meandered or rotated on the sphere, oscillating in the anterior-posterior direction of the cell body (Fig. 5e). By subtracting the rotational speed of the cell body from the trajectories of each dot, the trajectories in a rotating coordinate system that co-rotated with *Chlamydomonas* were determined. By plotting the velocity vectors of the membrane flow at each point of the trajectories, a planar map of the membrane flow field as observed from *Chlamydomonas* was obtained (Fig. 5f). Rapid southward flow occurs at two locations on the sphere (Φ=–90 and 90 degrees), and at positions 90 degrees away from these, slower northward return flows are observed. Together with the east-west flows connecting the north-south flows, four vortices centered near the equator were identified. These results experimentally verified the four-vortex structure on the liposome membrane predicted by the theoretical model. Notably, domain motion was also observed in translationally swimming Chlamylipo (Fig. S3,4, Movie S13), but was not observed in liposomes without encapsulated cells (Fig. S5).

## Discussion and conclusion

In this study, we created “Chlamylipo” by encapsulating *Chlamydomonas* inside liposomes and discovered that as the internal *Chlamydomonas* beat their flagella, the entire liposome swam forward and exhibited phototaxis. Two membrane protrusions appeared on the liposome membrane of Chlamylipo in association with flagellar movement, repeating a cycle in which they moved backward and disappeared. Both the internal and external fluids of Chlamylipo exhibited rapid circular or back-and-forth motions at several tens of Hz, while slowly migrating north–south at approximately 4 Hz. The membrane domains on the liposome surface also underwent small back-and-forth motions, moving quickly southward and then slowly northward at approximately 4 Hz. From the perspective of the rotating *Chlamydomonas*, the membrane flow field was a circulating current containing four vortices, which could be reproduced using a simplified flow-field model.

In a low Reynolds number environment where Chlamylipo exists, microscopic objects must repeatedly undergo time-irreversible asymmetric deformations to achieve continuous center of mass movement. Chlamylipo fulfills this requirement by repeatedly forming two membrane protrusions at the front that move backward and then disappear, creating a time-irreversible asymmetric deformation cycle. It is considered that as the membrane protrusions push the surrounding water masses backward, Chlamylipo moves forward in the reaction. Most Chlamylipo did not swim forward, and among those that did swim, there was variation in swimming speed. No clear correlation was observed between the swimming speed and liposome diameter. In addition, Chlamylipo, which did not form membrane protrusions, did not move forward. Even for liposomes of the same size, variations in the length of the membrane protrusions and the distance they travel backward are presumed to affect the movement speed of Chlamylipo. By examining the correlations between factors other than the liposome diameter, such as the protrusion length, duration of protrusion, and movement speed, it should be possible to clarify the factors that determine the swimming speed. By doing so, we could increase both the proportion of forward-swimming Chlamylipo and their swimming speed, thereby improving the efficiency of cargo transport by increasing the percentage of forward-moving Chlamylipo and their forward velocity.

The internal and external liquids and membrane flow consisted of small back-and-forth movements at several tens of Hz and slower movements at approximately 4 Hz. Based on the frequencies of these movements, the small back-and-forth motion was attributed to flagellar movement, and the slower motion was considered to result from the cell rotation. The small back-and-forth movement occurred at a frequency similar to that of flagellar motion, whereas the slower movement had a period approximately twice that of the rotation. This can be explained by the fact that the flagellar surface passes over the membrane twice during one full rotation of *Chlamydomonas*. Because the internal liquid, membrane, and external liquid exhibit similar behaviors, it is believed that the internal liquid flow generated by the flagellar motion of *Chlamydomonas* is transmitted through viscous friction to the liposome membrane and external liquid, causing a periodic movement.

The internal liquid of the liposome is a three-dimensional closed space with no exchange with the outside; therefore, the water mass pushed backward inevitably returns to the front. Similarly, the liposome membrane is a two-dimensional closed space with no exchange with the outside; therefore, the membrane molecules pushed backward inevitably return forward again. Therefore, on the flagellar plane, which includes the anterior-posterior axis of the cell body and the two flagella, a backward flow occurs, whereas on the median plane perpendicular to this, a forward return flow is thought to occur. The circulatory flow generated in the internal liquid by flagellar movement is believed to induce membrane flow through viscous friction, which, in turn, causes a circulatory flow in the external liquid near the membrane.

The angular momentum generated by the oblique beating of the flagella of *Chlamydomonas* can be interpreted as being balanced by the reaction forces produced by the flows of the inner fluid, membrane, and outer fluid, allowing free-swimming *Chlamydomonas* to maintain an equivalent rotational speed. This suggests that the *Chlamydomonas* inside Chlamylipo exhibited phototaxis, regardless of whether forward swimming occurred. By calculating the velocity field in the Chlamylipo coordinate system, we found that *Chlamydomonas* formed a circulatory flow with four vortices in the flow field. The membrane flow on the liposome surface is a two-dimensional, incompressible, viscous flow constrained by a sphere. The southward flow generated by flagellar motion must circulate and return to its original position within the two-dimensional closed region of the sphere. In Chlamylipo, because the flagella contact the membrane at two points where downward flow occurs, an upward flow arises at the median plane to return to the original position, resulting in circulatory flow with four vortices. The fastest flow velocity reached 1500 μm/s, which is comparable to the movement speed of the flagellar tip during effective stroke. Fitting a fluid model simulating the flow on a sphere to the measured data reproduced the circulation with four vortices, capturing the velocity differences between the southward and northward currents. The total force required to generate a flow field equivalent to the measured values was 81.3 pN at both the points. Previous research [22] reported a value of approximately 30 pN, suggesting that these values are of the same order of magnitude. In this study, the flow of the inner and outer fluids could only be observed in a single cross-section, and the flow in the direction perpendicular to the screen could not be detected. Because the flows of the inner fluid, membrane, and outer fluid were recorded using different Chlamylipos, their temporal correlations could not be established.

Although the global membrane flow, as observed in *Chlamydomonas*, was reconstructed, the meridional and oblique flows necessary for generating rotation could not be detected in this study. Because the *Chlamydomonas* inside the Chlamylipo exhibits precession motion while tilting its rotation axis, it is believed that the oblique flow cannot be accurately detected. By simultaneously observing the flows of the inner fluid, membrane, and outer fluid, as well as the morphological changes of the flagella in three dimensions within the same Chlamylipo, and analyzing them after correcting for the precession motion of the cell body, it will become possible to understand the mechanism by which periodic membrane deformation determines the forward velocity and how oblique fluid motion generates a rotation.

In this study, we found that Chlamylipo swims forward with flagellar motion and shows phototaxis. By co-encapsulating various compounds, microparticles, or microorganisms as cargo, such as fluorescent beads used as tracers, it should be possible to transport the cargo to a target location by controlling the swimming direction with light. A more detailed understanding of membrane deformation and the flow of internal fluid, membrane, and external fluid associated with flagellar motion is expected to improve the efficiency of active drug delivery systems (DDS).

## Conflict of interest

The authors declare no conflict of interest.

## Author contributions

S.S., M.H., H.S., K.A. and T.K. designed the project. S.S., M.H., H.S. and K.A. performed the experiments and analyzed the data. S.H. and D.M conducted the fluid mechanical analysis. M.H., H.S., K.A., D.M., T.K. wrote and checked the manuscript.

## Data availability

The evidence data generated/analyzed in this study are included in this article. A preliminary version of this work, DOI: https://doi.org/10.64898/2026.03.11.711009, was deposited in the bioRxiv on March 12, 2026.

## References

[1] Allen, T. M., Cullis, P. R. Liposomal drug delivery systems: From concept to clinical applications. Adv. Drug Deliv. Rev. 65, 36–48 (2013). https://doi.org/10.1016/j.addr.2012.09.037 23036225 10.1016/j.addr.2012.09.037

[2] Sercombe, L., Veerati, T., Moheimani, F., Wu, S. Y., Sood, A. K., Hua, S. Advances and challenges of liposome assisted drug delivery. Front. Pharmacol. 6, 286 (2015). https://doi.org/10.3389/fphar.2015.00286 26648870 10.3389/fphar.2015.00286 PMC4664963

[3] Bastos-Arrieta, J., Revilla-Guarinos, A., Uspal, W. E., Simmchen, J. Bacterial biohybrid microswimmers. Front. Robot. AI 5, 97 (2018). https://doi.org/10.3389/frobt.2018.00097 33500976 10.3389/frobt.2018.00097 PMC7805739

[4] Singh, A. V., Hosseinidoust, Z., Park, B. W., Yasa, O., Sitti, M. Microemulsion-based soft bacteria-driven microswimmers for active cargo delivery. ACS Nano 11, 9759–9769 (2017). https://doi.org/10.1021/acsnano.7b02082 28858477 10.1021/acsnano.7b02082

[5] Dogra, N., Izadi, S., Vanderlick, T. K. Micro-motors: A motile bacteria based system for liposome cargo transport. Sci. Rep. 6, 29369 (2016). https://doi.org/10.1038/srep29369 27377152 10.1038/srep29369 PMC4932553

[6] Alapan, Y., Yasa, O., Schauer, O., Giltinan, J., Tabak, A. F., Sourjik, V., et al. Soft erythrocyte-based bacterial microswimmers for cargo delivery. Sci. Robot. 3, eaar4423 (2018). https://doi.org/10.1126/scirobotics.aar4423 33141741 10.1126/scirobotics.aar4423

[7] Le Nagard, L., Brown, A. T., Dawson, A., Martinez, V. A., Poon, W. C. K., Staykova, M. Encapsulated bacteria deform lipid vesicles into flagellated swimmers. Proc. Natl. Acad. Sci. U.S.A. 119, e2206096119 (2022). https://doi.org/10.1073/pnas.2206096119 35969733 10.1073/pnas.2206096119 PMC9407364

[8] Foster, K. W., Saranak, J., Patel, N., Zarilli, G., Okabe, M., Kline, T., et al. A rhodopsin is the functional photoreceptor for phototaxis in the unicellular eukaryote *Chlamydomonas*. Nature 311, 756–759 (1984). https://doi.org/10.1038/311756a0 6493336 10.1038/311756a0

[9] Ueki, N., Ide, T., Mochiji, S., Kobayashi, Y., Tokutsu, R., Minagawa, J., et al. Eyespot-dependent determination of the phototactic sign in *Chlamydomonas reinhardtii*. Proc. Natl. Acad. Sci. U.S.A. 113, 5299–5304 (2016). https://doi.org/10.1073/pnas.1525538113 27122315 10.1073/pnas.1525538113 PMC4868408

[10] Ueki, N., Wakabayashi, K. Phototaxis assay for *Chlamydomonas reinhardtii*. Bio-protocol 7, e2356 (2017). https://doi.org/10.21769/BioProtoc.2356 34541103 10.21769/BioProtoc.2356 PMC8410251

[11] Leptos, K. C., Chioccioli, M., Furlan, S., Pesci, A. I., Goldstein, R. E. Phototaxis of Chlamydomonas arises from a tuned adaptive photoresponse shared with multicellular Volvocine green algae. Phys. Rev. E 107, 014404 (2023). https://doi.org/10.1103/PhysRevE.107.014404 36797913 10.1103/PhysRevE.107.014404 PMC7616094

[12] Bennett, R. R., Golestanian, R. A steering mechanism for phototaxis in *Chlamydomonas*. J. R. Soc. Interface 12, 20141164 (2015). https://doi.org/10.1098/rsif.2014.1164 25589576 10.1098/rsif.2014.1164 PMC4345482

[13] Lauga, E. The fluid dynamics of cell motility. Cambridge University Press. (2020). https://doi.org/10.1017/9781316796047

[14] Purcell, E. M. Life at low Reynolds number. Am. J. Phys. 45, 3–11 (1977). https://doi.org/10.1119/1.10903

[15] The cited paper was incorrect. Please replace Ref. 15 with the following: Che, Y., Song, X., Zhang, L. Engineering microalgae-based biohybrid robots for biomedical applications. Cell Biomater. 1, 100103 (2025). https://doi.org/10.1016/j.celbio.2025.100103

[16] Zhang, F., Li, Z., Chen, C., Luan, H., Fang, R. H., Zhang, L., et al. Biohybrid microalgae robots: Design, fabrication, materials and applications. Adv. Mater. 36, e2303714 (2024). https://doi.org/10.1002/adma.202303714 37471001 10.1002/adma.202303714 PMC10799182

[17] Gorman, D. S., Levine, R. P. Cytochrome f and plastocyanin: their sequence in the photosynthetic electron transport chain of *Chlamydomonas reinhardtii*. Proc. Natl. Acad. Sci. U.S.A. 54, 1665–1669 (1965). https://doi.org/10.1073/pnas.54.6.1665 4379719 10.1073/pnas.54.6.1665 PMC300531

[18] Pautot, S., Frisken, B. J., Weitz, D. A. Production of unilamellar vesicles using an inverted emulsion. Langmuir 19, 2870–2879 (2003). https://doi.org/10.1021/la026100v

[19] Pautot, S., Frisken, B. J., Weitz, D. A. Engineering asymmetric vesicles. Proc. Natl. Acad. Sci. U.S.A. 100, 10718–10721 (2003). https://www.pnas.org/doi/10.1073/pnas.1931005100 12963816 10.1073/pnas.1931005100 PMC196870

[20] Henle, M. L., Levine, A. J. Hydrodynamics in curved membranes: The effect of geometry on particulate mobility. Phys. Rev. E 81, 011905 (2010). https://doi.org/10.1103/PhysRevE.81.011905 10.1103/PhysRevE.81.011905 20365397

[21] Sakuma, Y., Taniguchi, T., Kawakatsu, T., Imai, M. Tubular membrane formation of binary giant unilamellar vesicles composed of cylinder and inverse-cone-shaped lipids. Biophys. J. 105, 2074–2081 (2013). https://doi.org/10.1016/j.bpj.2013.09.021 24209852 10.1016/j.bpj.2013.09.021 PMC3824544

[22] McCord, R. P., Yukich, J. N., Bernd, K. K. Analysis of force generation during flagellar assembly through optical trapping of free-swimming *Chlamydomonas reinhardtii*. Cell Motil. Cytoskeleton 61, 137–144 (2005). https://doi.org/10.1002/cm.20071 15887297 10.1002/cm.20071

