# Supplementary material for: Chlamylipo, a Chlamydomonas-in-liposome microswimmer: Self-propelled swimming and associated lipid membrane flow
Source: Biophys Physicobiol. 2026 May 26;23(2):e230019. doi: 10.2142/biophysico.bppb-v23.0019 (PMC13333194; doi:10.2142/biophysico.bppb-v23.0019)
Supplement: Supplementary file 1 — Supplementary Materials [file 23_e230019_1.pdf]

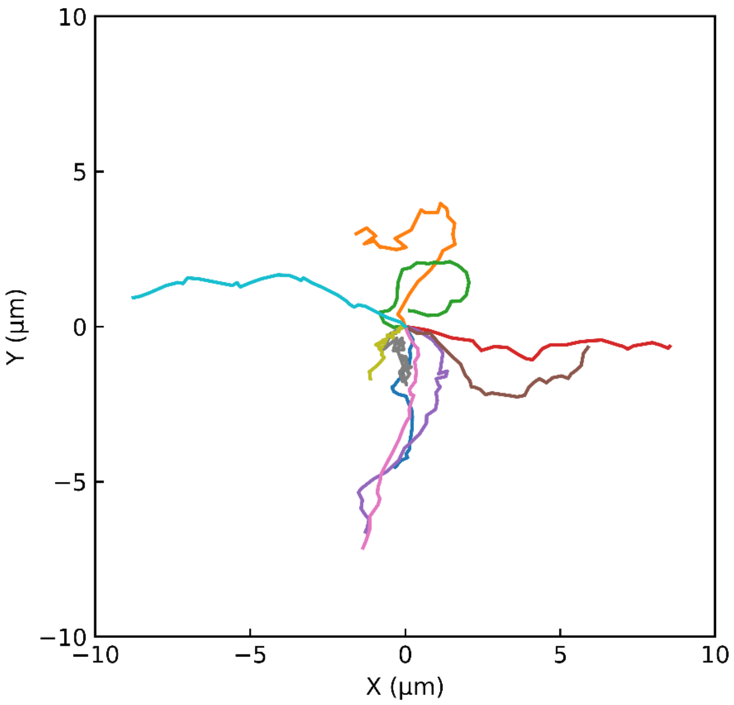

Fig. S1 Trajectory of Chlamylipos.
The movement of Chlamylipo was tracked for one second, starting from the origin as the initial position. The movements showed no directional patterns without photo stimulation (n=10).

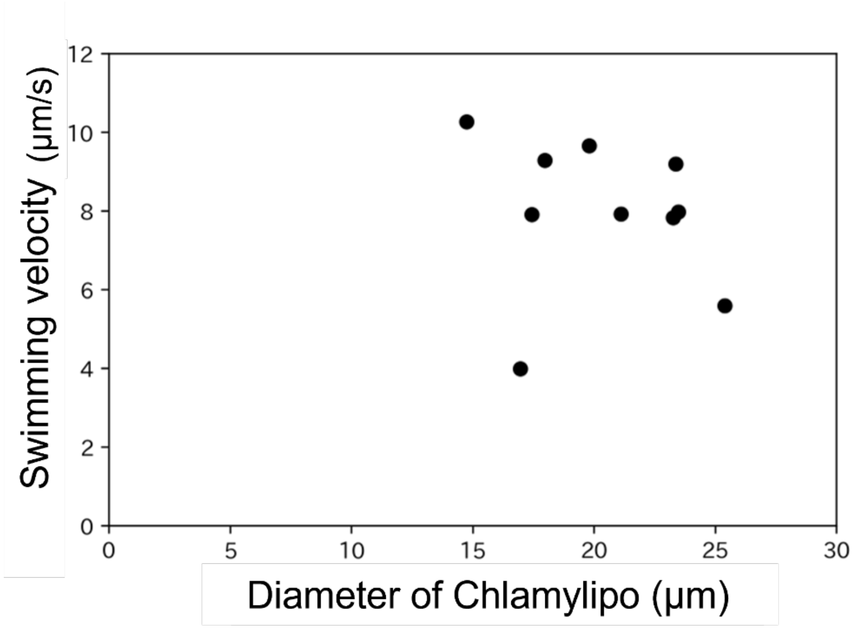

Fig. S2 Relationship between the diameter and swimming speed of Chlamylipo.
The diameter and movement speed of Chlamylipo are shown (n = 10). The movement speed was measured over 1 s. The diameter of the liposomes was determined as the average size of the liposomes in each frame during the movement.

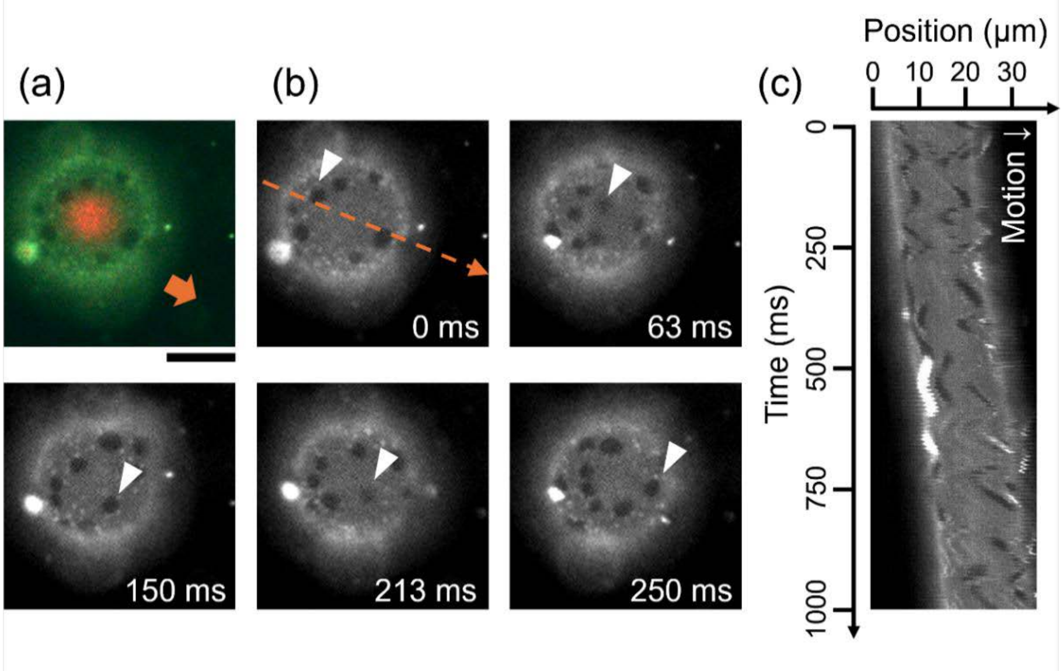

Fig. S3 Membrane fluidity of swimming phase separated Chlamylipo.

(a) Fluorescence color image of a liposome encapsulating *Chlamydomonas*. The green fluorescence represents 488 PE in the liposome membrane, and the red fluorescence is the autofluorescence of *Chlamydomonas*. The liposome deformed and moved as the internal *Chlamydomonas* swam. (b) Time-lapse images of the green fluorescence from (a). (c) Kymograph of the dashed area in (b). The white or gray regions indicate fluorescence from the liposome membrane, while the black regions represent domains or background. The domains moved back and forth accompanying the movement of Chlamylipo. Scalebar is 10 μm.

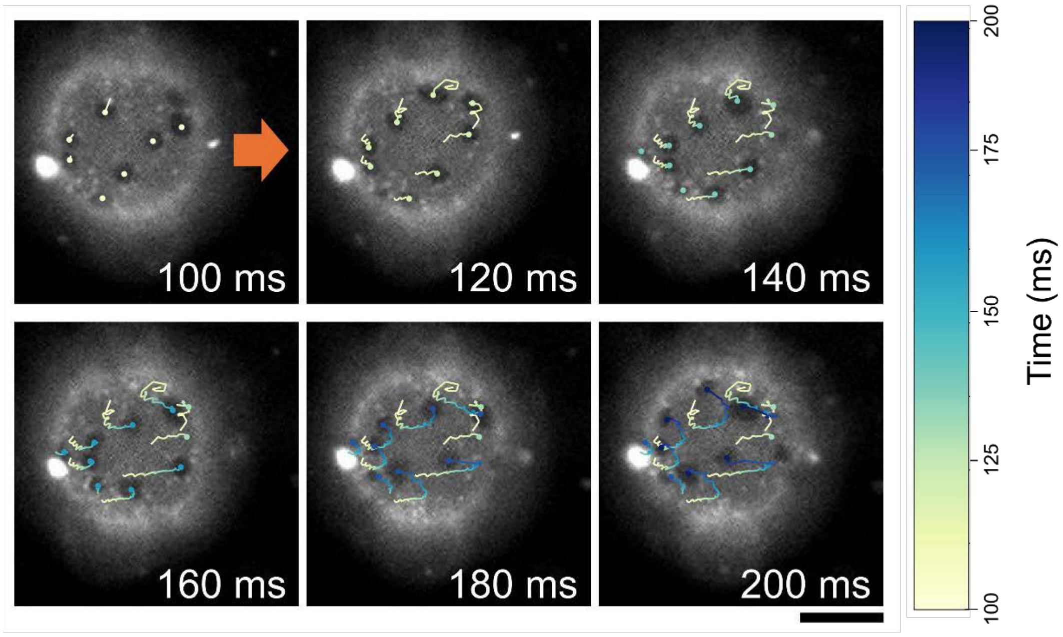

Fig. S4 Trajectory of the domain accompanying the movement of Chlamylipo.

Time-lapse images of the Chlamylipo domain. For Chlamylipo in Fig. S3, the movement of the domain between 100 and 200 ms was tracked. The arrows indicate the direction of Chlamylipo progression. The domain moved forward in front of the liposome and then backward. Scalebar is 10 μm.

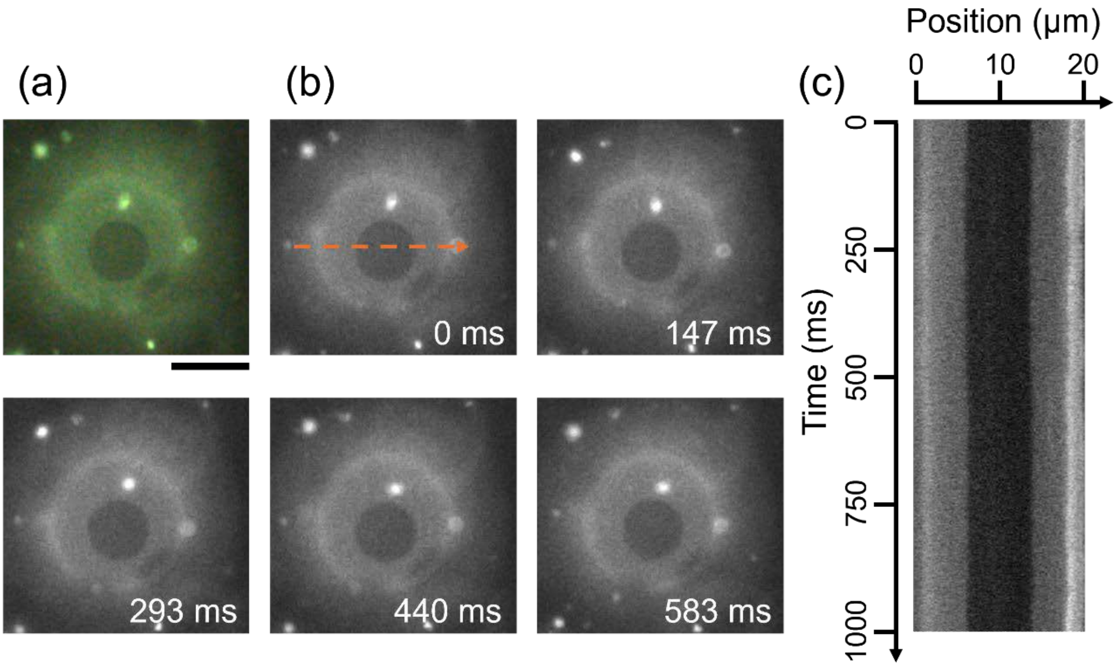

Fig. S5 Laterally phase-separated liposomes without encapsulated *Chlamydomonas.*

(a) Fluorescence color image of a phase-separated liposome. The green fluorescence corresponds to 488 PE incorporated into the liposome. Scalebar is 10 µm. (b) Time-lapse images of green fluorescence shown in (a). The dashed arrow indicates the line used to generate the kymograph in (c). The domains remained mostly stationary after this. (c) Kymograph taken along the dashed arrow in (b). The white or gray regions represent the fluorescence of the liposome membrane, while the black regions correspond to domains or background.

Movie S1. Chlamylipo was prepared using non-motile *pf18* mutants.
A liposome encapsulating the *pf18* mutant was prepared using the interface-crossing method, similar to that of the wild-type Chlamylipo. Because the cell lacks motility and does not induce membrane deformation, the liposome does not swim in the same manner as the cell. The video was played at real-time speed.

Movie S2. Chlamylipo encapsulating two cells.
Two *Chlamydomonas* cells were encapsulated within a single liposome, and their movements were not coordinated, and stable swimming was not observed. The video was played at real-time speed.

Movie S3. Phototactic swimming control of Chlamylipo.
Video overlaid with the trajectory of the center of gravity of Chlamylipo. The snapshots in Fig. 2a were obtained from the video. In addition to the change in swimming direction in response to the switching of green light, the trajectory exhibited a wavy pattern corresponding to the rotation of the cell. The video was recorded at 30 fps and played at real-time speeds.

Movie S4. Repetitive phototactic response of Chlamylipo.
The Chlamylipo shown in this video is identical to that presented in Fig. 2a and Movie S3. Chlamylipo successfully changed its swimming direction in response to repeated changes in the light direction. Directional control was achieved for over 16 turns, covering a total distance of approximately 1.2 mm. The video was played at a speed of 30 ×.

Movie S5. Periodic membrane deformation of Chlamylipo.
The Chlamylipo shown in this video is identical to that shown in Fig. 3a. Chlamylipo was prepared using fluorescent phospholipids and observed under dark-field illumination while being irradiated with excitation light. Periodic membrane deformations were observed using high-speed imaging at 600 fps. The video was played at 1/20× speed.

Movie S6. Internal fluid flow in Chlamylipo (m-plane).
The Chlamylipo shown in this video is identical to that shown in Fig. S4. Fluorescent beads were co-encapsulated within Chlamylipo to trace the fluid motion. High-speed imaging at 600 fps revealed periodic bead flows accompanying flagellar beating and cell rotation. This video shows the flow in the median plane (m-plane). The video was played at 1/100× speed.

Movie S7. Internal fluid flow in Chlamylipo (f-plane).
This video shows the bead flow in the flagellar plane (f-plane) under the same conditions as those in Movie S6. The video was played at 1/100× speed.

Movie S8. One-second observation of internal fluid flow.
The video shows the internal fluid flow recorded for 1 s. The video was played at 1/20× speed.

Movie S9. External fluid flows around Chlamylipo (m-plane).
The Chlamylipo shown in this video is identical to that presented in the bottom row of Fig. 4. Fluorescent beads were added to the external fluid to trace the motion. High-speed imaging at 600 fps revealed periodic bead flows accompanying cell movement. This video shows the flow in the m-plane. The video was played at 1/100× speed.

Movie S10. External fluid flows around Chlamylipo (f plane).
This video shows the bead flow in the flagellar plane (f-plane) under the same conditions as those in Movie S9. The video was played at 1/100× speed.

Movie S11. One-second observation of the external fluid flow.
The video shows the external fluid flow recorded for 1 s of the experiment. The video was played at 1/20× speed.

Movie S12. Membrane fluidity of Chlamylipo.
This movie shows the same Chlamylipo as shown in Fig. 5d. Lateral phase separation occurs in the liposomal membrane, allowing the observation of membrane fluidity through domain dynamics. High-speed imaging (300 fps) revealed domain displacement induced by the cell motility. Green indicates membrane fluorescence and red indicates chloroplast autofluorescence in *Chlamydomonas*. The video was played at 1/20× speed.

Movie S13. Membrane fluidity of swimming Chlamylipo.
This movie shows the observation of membrane fluidity during swimming Chlamylipo, which exhibits lateral phase

separation in its liposomal membrane upon exposure to a hypertonic solution. Similar to Chlamylipo (Fig. 5), back-and-forth motion of the domains accompanying flagellar beating was observed. Green indicates membrane fluorescence and red indicates chloroplast autofluorescence in *Chlamydomonas*. The video was played at 1/20x speed.
